# Violent victimization and revictimization in patients with depressive disorders: context characteristics, disclosure rates, and gender differences

**DOI:** 10.1186/s12888-022-04045-4

**Published:** 2022-06-16

**Authors:** C. Christ, M. M. de Waal, M. J. Kikkert, D. G. Fluri, A. T.F. Beekman, J. J.M. Dekker, D. J.F. van Schaik

**Affiliations:** 1 Amsterdam UMC, Department of Psychiatry, Amsterdam Public Health Research Institute, VU University Medical Center, Amsterdam, The Netherlands; 2 Department of Research and Innovation, GGZ inGeest Specialized Mental Health Care, P.O. Box 7057, 1007 MB Amsterdam, The Netherlands; 3 Department of Research, Arkin Mental Health Care, Amsterdam, The Netherlands; 4 PsyQ Mental Health Care, Zaandam, The Netherlands; 5 Department of Clinical Psychology, Faculty of Behavioral and Movement Sciences, Vrije Universiteit Amsterdam, Amsterdam Public Health research institute, Amsterdam, Netherlands

**Keywords:** Depression, Revictimization, Interpersonal violence, Sexual abuse, Physical assault, Threat, Mental illness, Disclosure

## Abstract

**Background:**

Depressed patients are prone to violent victimization, and patients who were victimized once are at increased risk to fall victim to violence again. However, knowledge on the context of victimization in depressed patients is lacking, and research identifying targets for prevention is urgently needed.

**Methods:**

This cross-sectional study explored context characteristics, disclosure rates and gender differences regarding violent victimization in 153 recently victimized depressed patients. Additionally, 12-month prevalence rates of repeat threat, physical assault, and sexual assault were examined, and gender differences were investigated using t-tests, Chi-square tests, and Fisher’s exact tests. Furthermore, logistic regression analyses were used to identify factors associated with repeat victimization.

**Results:**

Overall, depressed men were most often victimized by a stranger in public, and women by their partner or ex-partner at home. Regarding sexual assault, no gender differences could be examined. Patients were sexually assaulted most often by an acquaintance (50.0%) or stranger (27.8%). In all patients, the most recent incidents of threat (67.6%) and physical assault (80.0%) were often preceded by a conflict, and only a minority had been intoxicated prior to the assault. Notably, less than half of patients had disclosed their recent experience of threat (40.6%) and physical assault (47.1%) to their mental health caregiver. For sexual assault, this was only 20%. Less than one third of patients had reported their recent experience of threat (27.9%), physical assault (30.0%) and sexual assault (11.1%) to the police. 48.4% of patients had been victimized repeatedly in the past year, with no gender differences found. Only depressive symptoms and unemployment were univariately associated with repeat victimization, but not in the multiple model.

**Conclusions:**

The high prevalence of repeat victimization in depressed patients and their low disclosure rates stress the need to implement routine enquiry of victimization in mental health care, and to develop preventive interventions accounting for specific needs of men and women.

**Supplementary Information:**

The online version contains supplementary material available at 10.1186/s12888-022-04045-4.

## Background

Psychiatric patients are at risk to fall victim to violence (e.g., [1, 2]). Studies have reported 3 to 6-fold elevated odds of violent victimization among people with severe mental illness compared to the general population [3, 4]. Victimization is associated with increased psychiatric symptom severity [5, 6] and service utilization [7]. Research on victimization in psychiatric patients has focused primarily on patients with substance use disorders (e.g., [8]) and psychotic disorders [9, 10], and knowledge of victimization in depressed patients remains limited. A nationwide, Swedish study found that individuals with depressive disorder were 1.9 times more likely to experience future severe violent victimization than their siblings without mental illness [3]. In a cross-sectional study, Dutch depressed outpatients had experienced violent victimization (i.e., threat, physical assault, or sexual assault) 3.4 times more often than members of the general population [11]. The relationship between depressive symptoms and victimization appears to be bidirectional, since victimization may lead to depressive symptoms [12, 13] and depressive symptoms may lead to subsequent victimization [12, 14]. To date, however, the context of violent victimization experiences of patients with depressive disorder have received scant attention in both research and clinical practice.

Despite the high prevalence and detrimental effects of violence against psychiatric patients, victimization is not routinely assessed in mental health care [15, 16]. Consequently, victimization often remains undetected. Many patients do not report their experience of victimization to their caregiver [10, 17–20], with reported disclosure rates varying between 16.2% [17] and 67.7% [18]. Similarly, many patients do not report their experiences of victimization to the police, with disclosure rates ranging from 16.1% [19] to 58% [17, 18]. Importantly, research has shown that incidents perpetrated by someone close to the victim, such as a family member or partner, are reported to the police even less frequently than incidents committed by others [17, 21]. No study has yet determined to what degree depressed patients report their victimization experiences to the police or their mental health caregiver.

Victimization that remains undetected, cannot be acted upon by mental health professionals. This is of particular concern in light of high revictimization rates. People who have been victimized once, have a highly increased risk to become victimized again. Revictimization has mainly been studied among victims of childhood abuse, who are at risk of adult sexual and physical revictimization (e.g., [22, 23]). Although investigated less frequently, adult victims of violence are at increased risk of revictimization as well (e.g., [24]). Teasdale, Daigle and Ballard [25] demonstrated that this also holds for physically victimized patients with depressive disorders, of whom the majority were revictimized within the following year. Among victims, a small group experiences a disproportionate share of repeat victimization incidents [24, 26]. This already vulnerable group likely faces a vicious circle of repeat victimization and worsening symptoms, since revictimization leads to depressive symptoms and other mental health problems [27, 28].

To break this victimization-revictimization cycle in psychiatric patients, and depressed patients in particular, it is crucial to identify those with a history of violent victimization, and to gain insight into the context of their prior victimization experiences. Knowledge of assault characteristics, such as the perpetrator and location of the incident, may elucidate which situational circumstances place depressed patients at risk for victimization, and may facilitate the development of prevention programs. Although the responsibility for victimization lies solely with the perpetrator, preventive interventions should be developed to reduce depressed patients’ vulnerability to repeat victimization. So far, however, the context characteristics of victimization in depressed patients remain largely unclear. The only study specifically examining assault characteristics in depressed outpatients reported that the most recent incident of violent victimization was most often committed by a stranger (52.5%), and had most often occurred in public (47.5%) [11]. However, the authors did not differentiate between specific types of violent victimization, and did not examine whether assault characteristics differed between male and female patients.

A second pathway towards breaking the victimization-revictimization cycle may be to determine which victimized patients are particularly at risk for repeat victimization. In most non-clinical samples, female victims were at increased risk of repeated sexual victimization [29, 30] and overall violence [24] compared to males. Moreover, the association between mental disorder and victimization risk appears stronger for women than for men [2, 3, 18]. Apart from female gender, several other risk factors for revictimization have been identified, among which are younger age [22], unemployment, living alone [31], more mental health problems [25, 29, 30], and a history of violent offending [32]. It is not yet clear whether these factors are associated with repeat victimization in victimized depressed patients as well.

Up to now, there has been no detailed investigation into the context of victimization in male and female depressed patients. Gaining insight into their victimization experiences and identifying factors associated with repeat victimization may contribute to the development of prevention programs aimed at reducing revictimization in the women and men belonging to this vulnerable group. The current study was conducted in a clinical sample of depressed patients with a recent history of violent victimization, and its aims were fourfold. First, this study aimed to explore context characteristics of victimization by threat, physical assault, and sexual assault in victimized depressed outpatients, and to examine whether context characteristics differ between men and women. Second, this study aimed to determine the rates of disclosure of threat, physical assault, and sexual assault to one’s therapist and the police. Third, this study aimed to describe gender differences regarding the prevalence of repeat victimization by threat, physical assault, and sexual assault in the past 12 months in this victimized clinical sample. Based on previous evidence, we hypothesized that female depressed patients were repeat victims of overall violence and sexual violence more often than male patients [24, 29]. To our knowledge, no prior study has examined gender differences for repeated threats and physical assaults, specifically. We expected no gender differences regarding these specific types of repeat violence. Lastly, this study aimed to examine which demographic and clinical characteristics were associated with repeat victimization in depressed patients with a recent history of victimization. In accordance with prior research, we hypothesized that living alone, unemployment, a history of violent offending, and a higher level of depressive symptoms were associated with repeat victimization.

## Methods

### Design

This cross-sectional study utilized baseline data from a multicenter randomized controlled trial that investigates the effectiveness of an internet-based emotion regulation training aimed at reducing revictimization in recently victimized patients with depressive disorder. The study protocol was approved by the Medical Ethics Committee of the VU University Medical Center, and all participants provided informed consent. Details on the study design have been fully described elsewhere [33].

### Participants

Participants were recruited in six outpatient mental health centers in The Netherlands. Inclusion criteria were: (a) diagnosis of a depressive disorder or dysthymic disorder according to DSM-IV criteria, with or without a concurrent anxiety disorder other than Obsessive-Compulsive Disorder; (b) having been victim of violence (i.e., threat, physical assault, or sexual assault) in the previous three years; (c) having been assigned to evidence-based psychotherapy for depressive or anxiety disorder according to clinical practice guidelines; (d) access to a computer or tablet with internet connection; and (e) age of 18 years or older. Participants were excluded if they had insufficient understanding of the Dutch language or suffered from psychotic symptoms, bipolar disorder, substance dependency requiring immediate intervention in an addiction treatment center, or high suicide risk that required immediate intervention.

### Procedure

Recruitment took place between July 2016 and June 2019. All participants with depressive disorders enrolling for treatment were screened for a recent history of violent victimization and recruited by clinicians during regular intakes at the mental health centers. Subsequently, a telephone interview was conducted by a research assistant to assess all inclusion and exclusion criteria. After informed consent was obtained, the baseline assessment was completed via the internet.

### Measures

#### Demographic characteristics

Demographic characteristics were collected during the self-report assessment. Current DSM-IV diagnoses, as determined by clinicians during intake, were extracted from the electronic patient record.

#### Inclusion and exclusion criteria

The MINI International Neuropsychiatric Interview (MINI; version 5.0) [34, 35] was used to assess the presence of a major depressive disorder and dysthymic disorder at baseline. In addition, sections C, D, I, J, K, and L of the MINI were administered to check exclusion criteria by assessing the presence of suicidality, bipolar disorders, alcohol abuse or dependency, drug abuse or dependency, and psychotic disorders. In case of a severe substance use disorder according to the MINI, the researcher consulted with the patient’s clinician. Only if the substance use disorder was considered the primary diagnosis and immediate referral to an addiction treatment center was deemed necessary, the patient was excluded. The MINI is a structured, clinician-administered diagnostic interview based on the Diagnostic and Statistical Manual of Mental Disorders (Fourth edition; DSM-IV) and the International Classification of Diseases (Tenth revision; ICD-10). The MINI was administered by a trained research assistant.

#### Victimization

Victimization was measured with the Safety Monitor [36], developed by the Dutch Ministry of Security and Justice. The Safety Monitor is a self-report instrument that strongly resembles the International Crime Victims Survey (ICVS) [37] and is used by Statistics Netherlands (CBS) to measure victimization on a large scale. We only used the subsection assessing violent crimes, which are subdivided into threat, physical assault, and sexual assault. Threat was defined as being threatened to get hurt physically or sexually, without actual violence. Physical assault was defined as being physically hurt by someone deliberately, with or without the use of a weapon. Lastly, sexual assault was defined as any form of unwanted sexual touching against one’s will.

At the screening interview, three questions of the Safety Monitor were asked to determine the presence of victimization experiences in each of these categories in the previous three years. In addition, participants were asked to provide a brief description of these incidents. At the assessment, the Safety Monitor was used to examine whether and how often participants had experienced threat, physical assault, and sexual assault in the previous 12 months. The number of victimization experiences was only measured in the previous 12 months, and lifetime repeat victimization was not assessed. Repeat victimization was defined as having experienced 2 or more incidents of victimization in the past 12 months. When multiple types of violence had occurred simultaneously, each type was included. However, when a person had first been threatened with violence that was actually committed afterwards in the same situation, only the physical or sexual violence was included. Apart from the prevalence rates of victimization, the following context information of the most recent incident of each reported type of victimization in the previous 12 months was examined: the perpetrator (i.e., stranger, partner or ex-partner, relative, neighbor, or acquaintance [e.g., friend, colleague, other acquaintance]), location (i.e., at home, other’s home, in public [i.e., on the street, in a shop, in a restaurant, or in public transport], at work or school, or elsewhere), and whether the participant had reported the incident to the police (yes/no). Furthermore, we added three questions to the Safety Monitor to explore whether the participant had used alcohol or drugs prior to the incident (yes/no), whether a conflict had preceded the incident (yes/no), and whether the participant had disclosed the incident to his or her therapist (yes/no).

#### Violence perpetration

We extended the Safety Monitor with questions covering perpetration of violence. Participants were asked whether they had perpetrated threat, physical assault, and sexual assault in their lifetime and in the last 12 months.

#### Depressive symptoms

Depressive symptoms were assessed with the Inventory of Depressive Symptomatology – Self Report (IDS-SR) [38, 39]: a 30-item self-report questionnaire designed to measure depressive symptom severity in the past seven days. The IDS-SR includes all diagnostic DSM-IV criteria for major depressive disorder. All items are rated on a 4-point Likert scale from 0 to 3, and are equally weighted in the total score. The IDS-SR has highly acceptable psychometric properties [39, 40]. In this study, the internal consistency of the IDS-SR was good (α = 0.85).

### Statistical analysis

First, independent t-tests, Chi-square tests, and Fisher-Freeman-Halton exact tests were used to examine gender differences in demographic and clinical characteristics. Fisher-Freeman-Halton exact tests were used for unordered r x c tables with more than 20% of cells with an expected value of less than 5. For Chi-square tests, exact *p*-values were used. Second, we conducted Fisher’s exact tests and Fisher-Freeman-Halton exact tests to explore differences in context characteristics of the most recent incident of each type of victimization between men and women, and between participants who had been victimized repeatedly versus singularly in the past 12 months. Fisher’s exact tests were used for 2 × 2 tables with more than 20% of cells with an expected value of less than 5. Only patients who had been victim of that specific type of violence in the past 12 months were included in these analyses. Third, Fisher-Freeman-Halton exact tests were performed to examine whether an association existed between perpetrator type and location for each type of victimization for men and women separately. Fourth, independent t-tests, Chi-square tests, and Fisher’s exact tests were used to examine gender differences in prevalence of repeat victimization in the past 12 months. Fifth, we performed univariate logistic regression analyses to determine whether the following variables were associated with repeat victimization (any type; yes/no) in the past 12 months: age (continuous), living situation (living alone/not alone), employment status (employed/unemployed), partner status (partner/no partner), lifetime perpetration (yes/no), and depressive symptoms (continuous). Subsequently, variables with *p* < .1 in the univariate analyses were included in a multiple regression analysis to determine which variables were independently associated with repeat victimization. All statistical analyses were performed in SPSS Statistics 27.0., with statistical significance set at *p* < .05. For five participants, information on one or more demographic variables was missing (i.e., ethnicity, *n* = 1; employment status, *n* = 1; partner status, *n* = 3; violence perpetration, *n* = 1; treatment duration, *n* = 3). Pairwise deletion was used to handle these missing data. There were no other missing values.

## Results

### Sample characteristics

In total, 1616 patients were screened for eligibility, of whom 1159 (71.7%) did not have a recent history of violent victimization, 159 (9.8%) did not meet other inclusion criteria, and 145 (9.0%) declined participation. In total, 153 patients met inclusion criteria and provided informed consent. The mean age at baseline was 34.71 years (SD = 12.09; range = 19–66), and patients were mostly female (*N* = 103, 67.3%). An overview of the demographic and clinical characteristics of the participants, disaggregated by gender, is presented in Table 1. Women more often were unemployed than men (31.1% vs. 14.0%; *χ*^*2*^ = 4.903, *df* = 1, *p* = .030), and men more often had a drug use disorder than women (28.0% vs. 7.8%; *χ*^*2*^ = 11.192, df = 1, *p* = .001). No other significant gender differences were found.

All included participants had been violently victimized in the past three years. The distribution of the different types of victimization over the past three years is shown in Table 1. Physical violence was most frequently reported by both male (74.0%) and female (69.9%) patients, followed by threat (62.0% and 65.0%, resp.). Sexual violence was significantly more prevalent in women (35.0%) than men (8.0%) (*p* < .001). Women had more often experienced multiple types of victimization than men in the past three years (59.2% vs. 38.0%) (*p* = .047). 9.2% of all patients had been victim of all three types of victimization.

**Table 1:** Demographic and clinical characteristics and three-year victimization rates of male and female depressed patients

	Total (*N* = 153)	Men (*n* = 50)	Women (*n* = 103)	t/χ^2^	*p*
*Demographic characteristics*					
Age, *M* (SD)	34.71 (12.09)	37.28 (14.00)	33.47 (10.91)	1.693	.0﻿94
Western ethnicity^a^, *n* (%)	110 (71.9)	38 (76.0)	72 (69.9)	0.971	.341
Education^b^, *n* (%)				0.175	.941
Lower	20 (13.1)	7 (14.0)	13 (12.6)		
Medium	70 (45.8)	22 (44.0)	48 (46.6)		
High	60 (39.2)	18 (36.0)	42 (40.8)		
Unemployed^a^, *n* (%)	39 (25.5)	7 (14.0)	32 (31.1)	4.903	**.030**
Living alone, *n* (%)	37 (24.2)	14 (28.0)	23 (22.3)	0.590	.546
No partner^c^, *n* (%)	92 (60.1)	27 (54.0)	65 (63.1)	0.769	.472
*Clinical characteristics*					
Primary diagnosis, *n* (%)				8.233^d^	.101^d^
Depressive disorder	125 (81.7)	45 (90.0)	80 (77.7)		
Posttraumatic Stress Disorder	11 (7.2)	0 (0)	11 (10.7)		
Panic disorder	6 (3.9)	1 (2.0)	5 (4.9)		
Social phobia	4 (2.6)	1 (2.0)	3 (2.9)		
Generalized anxiety disorder	4 (2.6)	2 (4.0)	2 (1.9)		
Anxiety disorder not otherwise specified	3 (2.0)	1 (2.0)	2 (1.9)		
Depressive symptoms, *M* (SD)	41.90 (11.35)	40.10 (11.42)	42.77 (11.27)	-1.367	.174
Alcohol use disorder, *n* (%)	29 (18.9)	14 (28.0)	15 (14.6)	3.956	.052
Drug use disorder, *n* (%)	22 (14.4)	14 (28.0)	8 (7.8)	11.192	**.001**
Lifetime perpetrator of violence^a^, *n* (%)	77 (50.3)	29 (58.0)	48 (46.6)	2.103	.167
Past-year perpetrator of violence^a^, *n* (%)	19 (12.4)	10 (20.0)	9 (8.7)	4.135	.064
*Three-year victimization rates*					
Threat, *n* (%)	98 (64.1)	31 (62.0)	67 (65.0)	0.136	.723
Physical assault, *n* (%)	109 (71.2)	37 (74.0)	72 (69.9)	0.276	.704
Sexual assault, *n* (%)	40 (26.1)	4 (8.0)	36 (35.0)	12.663	**< .001**
Number of types, *n* (%)				6.119	**.047**
Victim in 1 category	73 (47.7)	31 (62.0)	42 (40.8)		
Victim in 2 categories	66 (43.1)	16 (32.0)	50 (48.5)		
Victim in 3 categories	14 (9.2)	3 (6.0)	11 (10.7)		

^a^Missing value for one participant (overall *N* = 152; men *n* = 49, women *n* = 103)

^b^Missing value for three participants (overall *N* = 150; men *n* = 47, women *n* = 103)

^c^Missing value for three participants (overall *N* = 150; men *n* = 48, women *n* = 102)

^d^Fisher-Freeman-Halton test

### Context characteristics of victimization

#### Threat

In victims of threat (*N* = 68), a significant association was found between victim gender and perpetrator type (*p* < .001), and between gender and incident location (*p* = .004) of the most recent incident in the past 12 months (see Table 2). In addition, there was a significant association between perpetrator and location of threat in both men (Fisher-Freeman-Halton = 28.407, *p* = .004) and women (Fisher-Freeman-Halton = 32.185, *p* < .001). Men were most often threatened by strangers (63.6%), which most often occurred in public (78.6%). Women were most often threatened by their (ex-)partner (45.7%), which mostly occurred at the victim’s home (66.7%). Threat by an acquaintance in men took place either in public (50%) or elsewhere (50%); in women, contrastingly, this most often occurred at work or school (50%) or at home (25%). Men had been significantly more often intoxicated during the incident (18.2%) compared to women (2.2%) (*p* = .035). No other significant gender differences were found. Of all patients, 67.6% reported that a conflict had preceded the most recent incident of threat.

People who had been victimized repeatedly in the past year were more often threatened by an (ex)partner (38.3%) and less often by a stranger (26.7%) compared to people who had been threatened once in the past year ([ex]partner 0%, stranger 62.5%; Fisher-Freeman-Halton test = 8.617, *p* = .034). No other significant differences were found regarding context characteristics of threat of violence between repeated victims and singular victims.

**Table 2:** Characteristics of the most recent incident of threat, physical assault, and sexual assault in recently victimized depressed patients

	Threats				Physical assaults				Sexual assaults
	Total (*n* = 68)	Men (*n* = 22)	Women (*n* = 46)		Total (*n* = 50)	Men (*n* = 16)	Women (*n* = 34)		Total^a^ (*n* = 18)
	%	%	%	*p*	%	%	%	*p*	%
**Perpetrator**				**< .001**^**b**^				**.020**^**b**^	
Stranger	30.9	63.6	15.2		20.0	37.5	11.8		27.8
(Ex-)Partner	33.8	9.1	45.7		38.0	31.3	41.2		16.7
Relative	11.8	4.5	15.2		20.0	0.0	29.4		5.6
Neighbor	5.9	4.5	6.5		10.0	18.8	5.9		0
Acquaintance	17.6	18.2	17.4		12.0	12.5	11.8		50.0
**Location**				**.004**^**b**^				.054^b^	
At home	41.2	13.6	54.3		42.0	25.0	50.0		27.8
Other’s home	4.4	4.5	4.3		14.0	6.3	17.6		16.7
In public	32.4	59.1	19.6		30.0	56.3	17.6		33.3
At work or school	8.8	9.1	8.7		10.0	6.3	11.8		11.1
Other	13.2	13.6	13.0		4.0	6.3	2.9		11.1
**Intoxicated**				**.035**^**c**^				.311^c^	
Yes	7.4	18.2	2.2		10.0	18.8	5.9		22.2
No	92.6	81.8	97.8		90.0	81.3	94.1		77.8
**Conflict prior to incident**				.407^c^				1.000^c^	
Yes	67.6	59.1	71.7		80.0	81.3	79.4		22.2
No	32.4	40.9	28.3		20.0	18.8	20.6		77.8

^a^ No gender differences could be examined due to small sample size (women *n* = 15, men *n* = 3)

^b^ Fisher-Freeman-Halton test

^c^ Fisher’s exact test

#### Physical assault

In victims of physical assault (*N* = 50), a significant association was found between victim gender and perpetrator type (*p* = .020) (see Table 2). Male patients were most often assaulted by a stranger (37.5%) or (ex-)partner (31.3%), and female patients by their (ex-)partner (41.2%) or relative (29.4%). A significant association between perpetrator and location of physical violence was found in women (Fisher-Freeman-Halton = 34.896, *p* < .001), but not in men (*p* = .100). Women were most often assaulted by their (ex-)partner, which mostly occurred at home (57.1%) or someone else’s home (28.6%), or by a relative, which mainly took place at the respondent’s home (90.0%). No other gender differences were found. A minority of victimized patients (10.0%) had used alcohol or drugs prior to being assaulted. 80.0% of patients reported that a conflict had preceded the most recent incident of physical assault.

People who had been victimized repeatedly in the past year were less often physically assaulted by a stranger (10.5%) compared to people who had been physically assaulted once in the past year (50.0%), and were more often assaulted by an ex-partner or relative (Fisher-Freeman-Halton test = 8.417, *p* = .049). In addition, repeated victims had been intoxicated significantly less often during the most recent incident of physical assault (2.6% vs. 33.3%) compared to patients who had been victimized once in the past year (Fisher’s Exact *p* = .009). No other significant differences in context characteristics were found.

#### Sexual assault

In total, 18 patients had been victims of sexual violence (11.8%). Due to the small number of male victims (*n* = 3), it was not possible to examine gender differences, and characteristics of sexual assault has only been described for the total group (see Table 2). For results stratified by gender, please see Supplemental Table 1. The most recent incident of sexual assault was most often perpetrated by an acquaintance (50.0%), followed by a stranger (27.8%). The incident most often occurred in public (33.3%) or at home (27.8%). Overall, most patients had not used alcohol or drugs prior to the incident (77.8%), and the incident had not been preceded by a conflict (77.8%). Due to the small sample size, it was not possible to examine differences in context characteristics between repeated and singular victims of sexual assault.

### Disclosure of victimization

For each type of victimization, a minority of patients had reported the most recent incident to the police. Regarding threat, 27.9% of victims had disclosed the most recent incident to the police. No significant association was found between perpetrator type and whether or not the incident had been reported. Regarding physical assault, less than one-third of victimized patients (30.0%) had reported the most recent incident to the police. A significant association was found between perpetrator type and whether or not the incident had been reported (Fisher-Freeman-Halton = 13.694, *p* = .004). Incidents of physical assaults perpetrated by a stranger had been reported in 60.0% of the cases, whereas incidents perpetrated by a relative or (ex-)partner had been reported in respectively 10.0% and 21.1% of the cases. Correspondingly, repeated victims had reported incidents of physical assault significantly less often to the police (21.1% vs. 58.3%) (Fisher’s Exact *p* = .027) than singular victims. Regarding threat, this association was not found. Lastly, the most recent incident of sexual assault had been reported to the police by 11.1% of sexual assault victims. For threat and physical assault, no significant differences were found between men and women. Regarding disclosure of sexual assault, no gender difference could be examined due to the small number of victims.

Disclosure to a therapist was determined only for those patients who had been in mental health treatment over the entire year prior to the assessment (*N* = 62; 40.5%). Of those patients, 62.9% (*n* = 39) had been violently victimized during that year. Of victims of physical assault (*n* = 17), less than half (47.1%) had disclosed the most recent victimization incident to their therapist. Of victims of threat (*n* = 32), 40.6% had discussed the incident with their therapist; for victims of sexual victimization (*n* = 5), this was 20%. No gender differences could be examined due to small sample sizes.

**Table 3:** Repeat victimization rates over the past 12 months in male and female depressed patients with a recent history of victimization

	Total (*N* = 153)	Men (*n* = 50)	Women (*n* = 103)	t/χ^2^	*p*
Violent victimization (any type)					
Victimized 0 times, *n* (%)	54 (35.3)	19 (38.0)	35 (34.0)	1.385	.497
Victimized 1 time, *n* (%)	25 (16.3)	10 (20.0)	15 (14.6)		
Victimized ≥ 2 times, *n* (%)	74 (48.4)	21 (42.0)	53 (51.5)		
Number of incidents, *M* (SD)	5.94 (18.02)	5.42 (21.23)	6.21 (16.31)	-0.255	.799
Threat					
Victimized 0 times, *n* (%)	85 (55.6)	28 (56.0)	57 (55.3)	0.121	.936
Victimized 1 time, *n* (%)	14 (9.2)	4 (8.0)	10 (9.7)		
Victimized ≥ 2 times, *n* (%)	54 (35.3)	18 (36.0)	36 (35.0)		
Number of incidents, *M* (SD)	4.20 (15.57)	4.48 (21.14)	4.08 (12.11)	0.149	.881
Physical assault					
Victimized 0 times, *n* (%)	103 (67.3)	34 (68.0)	69 (67.0)	1.000	.594
Victimized 1 time, *n* (%)	23 (15.0)	9 (18.0)	14 (13.6)		
Victimized ≥ 2 times, *n* (%)	27 (17.6)	7 (14.0)	20 (19.4)		
Number of incidents, *M* (SD)	1.51 (5.34)	0.78 (1.96)	1.87 (6.35)	-1.599	.112
Sexual assault					
Victimized 0 times, *n* (%)	135 (88.2)	47 (94.0)	88 (85.4)	2.088	.335^a^
Victimized 1 time, *n* (%)	8 (5.2)	1 (2.0)	7 (6.8)		
Victimized ≥ 2 times, *n* (%)	10 (6.5)	2 (4.0)	8 (7.8)		
Number of incidents, *M* (SD)	0.23 (0.73)	0.16 (0.77)	0.26 (0.71)	-0.811	.419

^a^ Fisher-Freeman-Halton test

### Repeat victimization

**Fig. 1:**
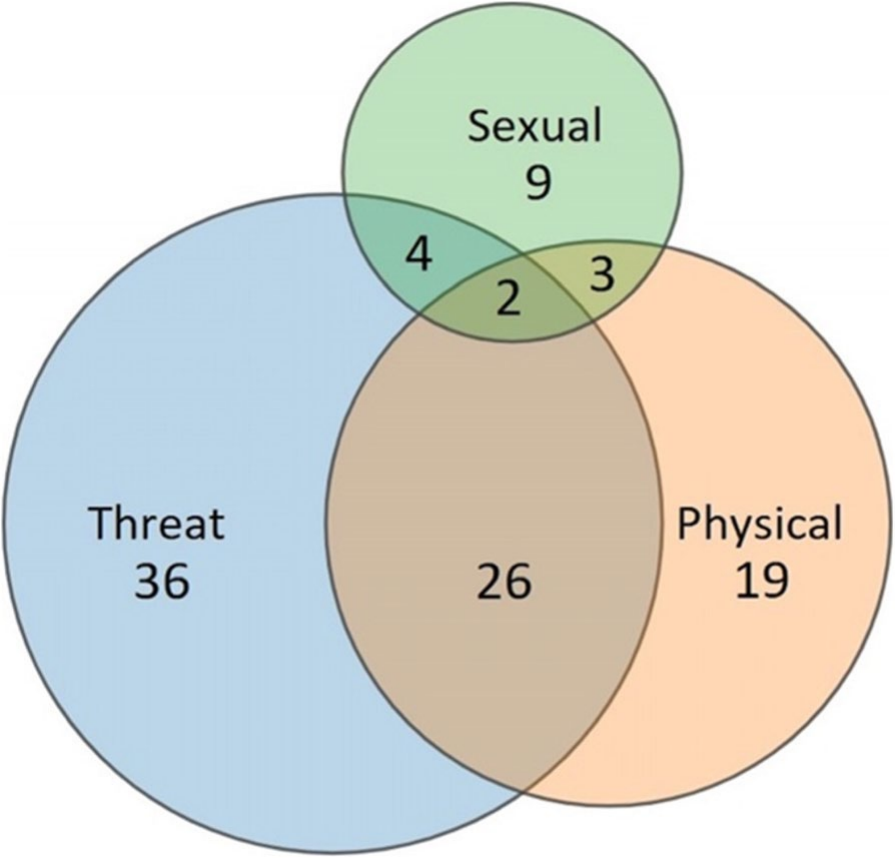
Distribution of and overlap between the reported types of violence (i.e., threat, physical assault, and sexual assault) in depressed patients who had been victimized in the past 12 months (*N* = 99)

Almost half (48.4%) of all patients had been victimized repeatedly in the past year. No significant gender differences regarding the prevalence of past-year repeat victimization were found for any type of victimization (see Table 3). For each type of victimization, patients had been more often victimized repeatedly than singularly in the past year. The average total number of incidents experienced by all patients in the past year was 5.95 (SD = 18.02; median = 1; range = 0-150; IQR = 4). The distribution of violent victimization incidents was highly skewed: patients who had been victimized five or more times in the past year accounted for only 22.9% of the sample, but had experienced 86.5% of all victimization incidents. Repeated threats had occurred in 35.3% of patients, with an average number of incidents of 4.21 (SD = 15.57; median = 0; range = 0-150; IQR = 3). Repeat physical assault and sexual assault were reported by respectively 17.6% and 6.5%, with the average number of incidents reported in the total sample being respectively 1.52 (SD = 5.34; median = 0; range = 0–50; IQR = 1) and 0.23 (SD = 0.73; median = 0; range = 0–5; IQR = 0). 35.4% of patients had experienced multiple types of violence in the past 12 months. Figure 1 shows the distribution of the three types of violence experienced by the *N* = 99 victims of past-year violence, including overlap between two or three types of victimization. Results demonstrated a substantial overlap between physical assault and threat, as *n* = 26 (26.3%) had experienced both types of victimization in the past year.

Univariate regression analyses revealed that unemployment (OR = 2.332 [95% CI 1.099–4.951]; *p* = .027) and depressive symptom severity (OR = 1.032 [95% CI 1.002–1.062]; *p* = .036) were positively associated with repeat violent victimization in the past 12 months. As shown in Table 4, none of the other examined variables (i.e., age, living situation, partner status, and lifetime history of violent offending) were significantly associated with repeat victimization (each *p* > .10). Hence, only employment status and depressive symptoms were included in the multiple regression model, which was overall significant (*χ*^*2*^ = 8.758, *df* = 2, *p* = .013; *R*^*2*^ = 0.075). As shown in Table 5, however, neither employment status, nor depressive symptoms were independently associated with past-year repeat victimization in the full model. The Hosmer and Lemeshow test indicated a proper fit to the data (*p* = .485).

**Table 4:** Results of univariate logistic regression analyses for associations with repeat violent victimization in depressed patients (*N* = 153)

	OR	95% CI	*p*
Age in years	1.003	0.977–1.029	.848
Living situation	1.560	0.740–3.290	.243
Employment status^a^	**2.332**	**1.099–4.951**	**.027**
Partner status^b^	1.545	0.796–3.001	.199
Lifetime perpetration^a^	1.304	0.689–2.466	.415
Depressive symptoms	**1.032**	**1.002–1.062**	**.036**

^a^Missing value for one participant (overall *N* = 152)

^b^Missing value for three participants (overall *N* = 150)

**Table 5:** Results of multiple logistic regression analysis for associations with repeat violent victimization in depressed patients (*N* = 153)

	OR	95% CI	*p*
Employment status^a^	2.075	0.963–4.471	.062
Depressive symptoms	1.030	0.999–1.061	.058

^a^Missing value for one participant (overall *N* = 152)

## Discussion

The first objective of this study was to explore the context characteristics of victimization in depressed patients subjected to violence and to compare these between men and women. Regarding recent threat and physical assault, the type of perpetrator differed significantly between men and women. Depressed men had most often been victimized by a stranger in public, whereas women had most often been victimized by an (ex-)partner at home. These results largely accord with gender differences found in clinical [15, 19, 41] and population-based samples [42–44]. For male depressed patients, reported victimization characteristics were in line with those among victimized men in the Dutch general population. Depressed women, however, were relatively far more likely to become victimized by their (ex-)partner and in their home than women in the general population [45]. Regarding sexual assault, no gender differences could be examined. In the total group of sexual assault victims, patients had most often been victimized by an acquaintance or a stranger, which most often took place at home or in public. Compared to patients with dual diagnosis, depressed patients were relatively far more often sexually assaulted by an acquaintance (40% vs. 14.8%) [19].

Our results showed that incidents of threat and physical violence in repeat victims of violence had been committed by a stranger less often than incidents in one-time victims, and more often by a partner, relative, or other close acquaintance. Hence, repeat victimization in depressed patients appears to mainly occur in the domestic context of patients, rather than in contact with strangers.

Only a minority of depressed victims had used alcohol or drugs prior to their most recent experience of threat (7.4%), physical assault (10.0%) and sexual assault (22.2%). Regarding threats, men had used alcohol or drugs more often (18.2%) than women (2.2%). Many studies have pointed out alcohol and substance use as correlates or risk factors for victimization in clinical (e.g., [25, 46, 47]) and population-based samples (e.g., [22, 30, 48, 49]). However, our results show that victimization by threat and physical assault had only rarely been preceded by the victim’s substance use−particularly in women, and also particularly in repeated victims, who had been intoxicated less often during the most recent incident of physical assault than singular victims (2.6% vs. 33.3%). Because the low proportion of victims who had been intoxicated during the most recent incident was quite unexpected, we performed post-hoc univariate logistic regression analyses to determine associations between presence of a substance use disorder and repeat victimization. Results showed that neither any alcohol use disorder, nor any drug use disorder were significantly associated with overall repeat victimization in our sample of depressed patients (OR = 1.180 [95% CI 0.526–2.651], *p* = .688 and OR = 0.703 [95% CI 0.281–1.757], *p* = .451, respectively). These findings provide a first indication that substance use may not play an important role in repeat victimization of depressed patients. However, since only depressed patients with a secondary diagnosis of substance use disorders were included in the current study, results may not be generalizable to depressed patients with more severe substance use disorders.

Importantly, our study shows that in most patients a conflict had preceded their experience of physical assault (80.0%) and threat (67.6%). No differences were found between men and women. Sexual assault had been preceded by a conflict far less often (22.2%). These results largely correspond with previous evidence that high interpersonal problems and low social support are associated with victimization [50–52]. This may be particularly relevant for patients with depressive disorders, who have been demonstrated to report high levels of interpersonal problems [53]. Future research is required to determine whether interpersonal problems are a mechanism in the relationship between depression and violent victimization.

The second objective of this study was to establish disclosure rates of victimization among victimized depressed patients. Our study shows that less than one third of patients had reported the most recent incident of threat (27.9%) and physical assault (30.0%) to the police. For sexual assault, this was only 11.1%. Our findings correspond with previous research indicating that sexual assault is less likely to be reported to the police than physical assault [19, 54]. Depressed patients were less likely to report their experience compared to other psychiatric samples [17, 18] and victims in the Dutch general population [55]. In line with previous findings, patients reported incidents of physical assault perpetrated by an (ex-)partner or relative less often than incidents perpetrated by a stranger [17, 21].

Notably, only 20% of patients who had been in treatment during the previous year had disclosed their experience of sexual assault to their mental health therapist, which is low compared to other clinical samples [19, 20, 56]. Disclosure rates of threat (40.6%) and physical assault (47.1%) were largely similar to those in most clinical samples [18, 20, 56]. We did not assess reasons not to disclose, but previous research suggests that the lack of enquiry by caregivers plays an important role [57]. It is important to emphasize that these low disclosure rates are likely to be an overestimation of the true disclosure rates in victimized, depressed patients, because our study could only include patients who were willing to disclose their victimization experiences to a researcher. Hence, patients not willing to disclose their victimization experiences to anyone were not included.

The third objective of this study was to describe the prevalence rates of repeat victimization in victimized depressed patients, and to examine whether these differ between male and female patients. Nearly half (48.4%) of our sample had repeatedly fallen victim to violence in the past year. The prevalence of repeat victimization was somewhat low compared to results from the only other study on repeat victimization among previously victimized depressed patients who were released from psychiatric hospital [25]. However, repeated victimization was more prevalent than singular victimization across all types. Contrary to our hypothesis and previous studies, we found no differences between men and women in the prevalence of repeat overall and sexual victimization [24, 29, 30]. Regarding sexual victimization, this contrasts the higher prevalence of non-repeat sexual victimization found in women compared to men in most clinical samples (e.g., [19, 41]) and the Dutch general population [45]. Consistent with our hypothesis and previous research on non-repeat victimization in clinical samples, we found no gender differences in prevalence of repeat threat and physical assault [4, 19, 41].

Lastly, we aimed to identify demographic and clinical factors associated with repeat victimization. Unexpectedly, only unemployment and depressive symptoms were weakly associated with past-year repeat victimization, and neither were independently associated with repeat victimization in the full model. Our results largely contrast most previous research identifying younger age [22], unemployment, living alone [31], violent offending [32], and higher levels of depressive and psychiatric symptoms [25, 29, 58] as risk factors for repeat victimization. However, these studies were mostly conducted among non-clinical samples. In the current study, both repeat and non-repeat victims were diagnosed with a depressive disorder and generally had a high symptom severity. Therefore, the variation may have been too small to detect a significant relationship. This is reflected by the high levels of depressive symptoms in the total sample, with relatively low standard deviations. Furthermore, the lack of identified predictors may partially be explained by the overlap between risk factors for non-repeat victimization and repeat victimization [31]. As all patients had been victimized in the past three years, they likely shared certain characteristics that made them prone to their previous victimization experience, and also increased their risk of subsequent repeat victimization. This is especially likely given the recency of violent victimization in our entire sample, which increases the likelihood that those factors have remained unchanged. This explanation is in line with an influential criminological perspective on revictimization: the risk heterogeneity perspective, which suggests that fundamental differences between crime victims and non-victims account for both initial and repeat victimization experiences of the first group (e.g., [59–61]).

### Limitations

To our knowledge, this study was the first to provide a detailed investigation of the context characteristics and disclosure rates of victimization in male and female victimized depressed patients and to examine the prevalence of repeat violent victimization in this group. However, this study is not without limitations. First, due to its cross-sectional design, no conclusions on causality can be drawn. Second, past-year repeat victimization was measured retrospectively, without controlling for lifetime repeat victimization. Accordingly, patients with a less recent history of repeat victimization were considered non-repeat victims, which may have distorted our findings. Third, our sample size was relatively small. Therefore, power to detect significant associations was limited, and some analyses may have yielded potentially biased estimates. In addition, we were unable to investigate a large number of factors putatively associated with repeat victimization. The low amount of explained variance in our statistical model indicates that other factors not included in the current study play a role in predicting repeat victimization in victimized depressed patients. Longitudinal research is needed to identify risk factors for repeat victimization in this group, including both demographic and clinical characteristics, such as childhood abuse [22] and interpersonal problems [51]. Given the small group reporting a past-year incident of sexual assault, we were not able to compare the context characteristics of sexual assault between men and women. Fourth, our sample concerned a convenience sample of treatment-seeking patients with high levels of depressive symptom severity, and the generalizability of our results to other samples may be limited. Fifth, our sample only included patients who were willing to disclose their experiences of victimization to a researcher; therefore, people who were unwilling or unable to share their experiences are not represented in this sample. This selection bias may have distorted our findings. Lastly, victimization was measured using a self-report questionnaire, which may be subject to memory bias.

### Clinical implications

The high prevalence of repeat victimization in victimized women and men with depressive disorders highlights the importance of identifying victimized patients and developing preventive interventions aimed at reducing their revictimization risk. Moreover, our findings regarding context characteristics stress the need for separate prevention programs tailored for men and women. In depressed men, victimization primarily occurred in contact with strangers in public. Thus, enhancing their awareness of potentially dangerous situations in public and recognizing aggression in others may help reduce their revictimization risk. In depressed women, victimization by threat and physical assault primarily occurred in contact with their (ex-)partners. Prevention strategies may aim to enhance their awareness of potentially dangerous situations and behavioral patterns in unhealthy relationships. Furthermore, advocacy interventions and cognitive behavior therapy may effectively reduce physical and psychological intimate partner revictimization [62–64]. Regarding sexual assault, increasing depressed patients’ awareness of risky situations and assertiveness in contact with acquaintances and strangers may help preventing repeat victimization. In general, given that the large majority of both men and women experienced a conflict preceding their experiences of threat and physical assault, strengthening interpersonal and conflict management skills appears useful for both groups. Prior research has shown that interventions aimed at enhancing interpersonal skills, risk awareness, and healthy relationship behavior effectively prevented victimization in dual diagnosis patients [65] and revictimization in adolescents [66, 67]. To date, it remains unknown whether and how revictimization in depressed patients may be effectively reduced. This study is part of a larger research that contributes to this knowledge gap by investigating the effectiveness of an internet-based emotion regulation training in reducing revictimization [33].

Importantly, the low disclosure rates found in this study underline the urgent need for mental health services to actively and routinely screen patients for victimization experiences. First, the detection of a history of victimization is essential to identify those at risk for repeat victimization and to provide them suitable care to cope with their experiences and prevent future revictimization. Second, clinicians’ detection of ongoing, repeat victimization is of vital importance to fully comprehend the current situation and safety of their patients. After all, ongoing victimization may hamper treatment effects, particularly in the case of severe violence [62]. Therefore, mental health services should facilitate disclosure by implementing routine enquiry of victimization experiences and training mental health professionals in adequately addressing and responding to victimization disclosures [15, 68, 69].

## Conclusions

The high prevalence of repeat violent victimization in victimized, depressed patients and the low disclosure rates found in this study stress the need to implement routine enquiry of violent victimization in mental health care. In addition, efforts should be made to develop and investigate interventions targeting high-risk populations, while accounting for the specific needs of men and women.

## Supplementary Information

**Additional file 1.**

## Data Availability

The datasets generated during and analyzed during the current study are not publicly available due to the ongoing follow-up data collection, but are available from the corresponding author on reasonable request.
