# Supplementary material for: Violent victimization and revictimization in patients with depressive disorders: context characteristics, disclosure rates, and gender differences
Source: BMC Psychiatry. 2022 Jun 16;22:403. doi: 10.1186/s12888-022-04045-4 (PMC9202098; doi:10.1186/s12888-022-04045-4)
Supplement: Supplementary file 1 — Additional file 1. [file 12888_2022_4045_MOESM1_ESM.docx]

Supplemental Table 1. Characteristics of the most recent incident of sexual assault in recently victimized depressed patients.

|  | Total (*N* = 18) | Men (*N* = 3) | Women (*N* = 15) |
| --- | --- | --- | --- |
|  | % | % | % |
| **Perpetrator** |  |  |  |
| Stranger | 27.8 | 0.0 | 33.3 |
| (Ex-)Partner | 16.7 | 33.3 | 13.3 |
| Relative | 5.6 | 0.0 | 6.7 |
| Acquaintance | 50.0 | 66.7 | 46.7 |
| **Location** |  |  |  |
| At home | 27.8 | 33.3 | 26.7 |
| Other’s home | 16.7 | 0.0 | 20.0 |
| In public | 33.3 | 33.3 | 33.3 |
| At work/school | 11.1 | 33.3 | 6.7 |
| Other | 11.1 | 0.0 | 13.3 |
| **Intoxicated** |  |  |  |
| Yes | 22.2 | 0.0 | 26.7 |
| No | 77.8 | 100 | 73.3 |
| **Conflict prior to incident** |  |  |  |
| Yes | 22.2 | 33.3 | 20.0 |
| No | 77.8 | 66.7 | 80.0 |
